# Heat stress inhibits the proliferation and differentiation of myoblasts and is associated with damage to mitochondria

**DOI:** 10.3389/fcell.2023.1171506

**Published:** 2023-04-11

**Authors:** Jiawei Lu, Huixia Li, Debing Yu, Peng Zhao, Yuan Liu

**Affiliations:** ^1^ College of Animal Science and Technology, Nanjing Agricultural University, Nanjing, China; ^2^ College of Animal Science, Tibet Agriculture and Animal Husbandry University, Linzhi, Xizang, China

**Keywords:** heat stress, myoblasts, mitochondria, proliferation, differentiation

## Abstract

**Introduction:** Heat stress is harmful to the health of humans and animals, more and more common, as a consequence of global warming, while the mechanism that heat stress modulates skeletal development remains unknown. Hence, we conducted a model of heat stress *in vitro*.

**Methods:** We used Hu sheep myoblasts as the research object, real-time quantitative PCR (RT-qPCR) and western blot (WB) were conducted to detect the expression of mRNA and protein in heat-stressed myoblasts. The would-healing assay was used to detect the migration of myoblasts. The mitochondria were observed by a transmission electron microscope.

**Results:** mRNA and protein expression of HSP60 was significantly enriched in the heat-stressed myoblasts during proliferation and differentiation (*p* < 0.05). In our study, we indicated that heat stress enriched the intracellular ROS of the myoblasts (*p* < 0.001), leading to an increase in autophagy in the myoblasts to induce apoptosis. The results demonstrated that the protein expression of LC3B-1 and BCL-2 was significantly increased in myoblasts under heat stress during proliferation and differentiation (*p* < 0.05). Additionally, heat stress inhibited mitochondrial biogenesis and function and reduced the mitochondrial membrane potential and downregulated the expression of *mtCo2, mtNd1* and *DNM1L* (*p* < 0.05) in myoblasts during proliferation and differentiation. Consequently, heat stress inhibited the proliferation and differentiation of the myoblasts, in accordance with the downregulation of the expression of *PAX7, MYOD, MYF5, MYOG* and *MYHC* (*p* < 0.05). Moreover, heat stress also inhibited the cell migration of the myoblasts.

**Discussion:** This work demonstrates that heat stress inhibits proliferation and differentiation, and accelerates apoptosis by impairing mitochondrial function and promoting autophagy, which provides a mechanism to understand heat stress affects the development of the skeletal muscle.

## 1 Introduction

The intergovernmental panel on climate change (IPCC) reports that the annual average temperature and seasonal temperature have increased than the natural changes during the past 40 years (IPCC, 2021). The global warming occurs resulting from the emissions of the greenhouse gas, CO_2_, CH_4_, and N_2_O, which leads to increased temperature by 1°C–2°C on the surface of the earth (IPCC, 2021; Kumar et al., 2021). Heat stress is the summation of the non-specific response when the excessive temperature exceeds the self-thermoregulation of humans or animals (Biance, 1962). All thermostatic animals have a zone of thermal neutrality, and the animals would suffer from heat stress when the ambient temperature is higher than the upper limit of the isothermal zone, leading to a series of physiological and pathological adverse reactions in animals (Dantzer and Mormède, 1983). Heat stress harms growth performance and carcass quality, and enriches the maintenance cost of animals because of the increased health problems (Gonzalez-Rivas et al., 2020). Heat stress is classified as acute or chronic according to duration and severity (Petracci et al., 2015; Lu et al., 2017; Wang et al., 2017), and whether acute or chronic, has been linked to a decrease in growth rate, lower feed efficiency, weakened immune function, changes in the gastrointestinal microbiota and poor meat quality (Zaboli et al., 2019). In the terms of meat quality, broilers have pale muscle after acute or chronic heat stress, which is characterized by pale soft exudative meat (PSE-meat), the cause might be the negative influence of heat stress on aerobic metabolism and glycolysis of the meat (Wilhelm et al., 2010; Lara and Rostagno, 2013). In the terms of female reproduction, heat stress directly affects the concentration of follicle-stimulating hormone (FSH) and inhibin in the cow plasma and indirectly affects the growth of follicles (Roth et al., 2000). Additionally, heat stress reduces the contents of estradiol, aromatase activity and the level of luteinizing hormone (LH) receptor in the plasma and follicles of goats and rats, leading to low estrus and follicular quality, delayed ovulation and even no ovulation (Ozawa et al., 2005). 15%–30% of bovine oocytes induce apoptosis under high temperatures (Roth and Hansen, 2004).

Skeletal muscle is one of the most important tissues of the body and maintains movement and basal energy expenditure (Frontera and Ochala, 2015). Maternal heat stress during pregnancy not only impairs the muscle fiber hyperplasia of fetal piglets but also reduces the vascular density of the longissimus dorsi (LD) muscles, which is associated with the changes of genes modulating the development and metabolism of the skeletal muscles by RNA sequencing (Zhao et al., 2022). Heat stress initiates oxidative stress in skeletal muscle and systemic inflammatory response in white adipose tissue of beef cattle by altering the expression of genes related to the metabolic and immune pathways (Reith et al., 2022). The function of mitochondria is changed in the skeletal muscle of heat-stressed mice (Chen et al., 2022). In broilers, chronic heat stress decreases the carcass weight and total breast meat, the adverse impact of heat stress is correlated with the changes in the metabolic pathways of muscle and fat, which is not completely explicated by the decreased feed intake (Teyssier et al., 2022).

We have recently reported that rosmarinic acid accelerated the myogenic differentiation of C2C12 myoblasts and maintained the formation of myotubes in a heat-stressed condition (Chen et al., 2020). In this study, we explored the effects of heat stress (42°C) on mitochondrial biogenesis and function, as well as the proliferation, differentiation, apoptosis and regeneration in Hu sheep myoblasts to provide the mechanism reference for the impairment of heat stress on the growth and development of human muscles.

## 2 Materials and methods

### 2.1 Cell culture and hyperthermia treatment

According to the report, the primary myoblasts were isolated by enzyme digestion and differential adhesion methods (Deng et al., 2021). Specifically, the thigh muscle tissues of a newborn Hu sheep fetus were cut and chopped, then 0.1% collagenase I (Sigma-Aldrich, St. Louis, Missouri, United States) was used to digest the muscle tissues for 90 min at 37°C with a gentle shake every 5 min, then the muscle tissues were digested with 0.25% trypsin (Gibco, Grand Island, NY, United States) for 20 min at 37°C. After digestion, the tissues were filtered through a 100-μm filter and then centrifuged to collect the cell precipitation. The cells were resuspended with the growth medium (DMEM/F12 (Gibco, Grand Island, NY, United States) including 20% fetal bovine serum (Gibco, Grand Island, NY, United States), 10% horse serum (Gibco, Grand Island, NY, United States) and 1% penicillin/streptomycin (Gibco, Grand Island, NY, United States)) and cultured in a cell incubator of 5% CO_2_ at 37°C. The non-adherent cells were transferred into another culture dish every 2.5 h, and the pure myoblasts were obtained after two transfers. The growth medium was changed every 2 days, and the growth medium was changed into the differentiation medium (DMEM/F12 including 2% horse serum and 1% penicillin/streptomycin) after an 80% confluence of myoblasts to induce differentiation.

Establishment of a heat-stressed model of myoblasts *in vitro*: when the myoblasts reached 80% confluence, the myoblasts were transferred to a cell incubator of 5% CO_2_ at 42°C for 3 h to conduct a heat-stressed model at the proliferation phase; when the myoblasts differentiated into numerous myotubes, the myoblasts were transferred to a cell incubator of 5% CO_2_ at 42°C for 3 h to perform a heat-stressed model at the differentiation phase, and the temperature and time of heat stress conducted in this study were in the light of a previous study (Chen et al., 2020).

### 2.2 RNA extraction and real-time quantitative PCR

RNA of the myoblast was extracted by trizol (Invitrogen, Waltham, CA, United States). Then RNA was reversed and transcribed into cDNA by the HiScript III RT SuperMix for the qPCR kit (Vazyme, Nanjing, China). The total volume of each reaction hole was 20 μL including 2 μL cDNA for RT-qPCR. Subsequently, RT-qPCR was conducted using the ChamQ universal SYBR qPCR Master Mix (Vazyme, Nanjing, China) and the QuantStudio 5 Real-Time PCR System (Life Technologies, New York, NY, United States). The reaction procedures were 95°C for 30 s to amplify, the circulation reaction was 95°C for 10 s and 60°C for 30 s and 40 circulations, and the melt curve phase was 95°C for 15 s, 60°C for 60 s and 95°C for 10 s. The relative expression of genes was quantified by the 2^−ΔΔCt^ method and *GAPDH* was used as the control gene. The primers used in this study were presented in Supplementary Table S1.

### 2.3 Immunofluorescence and confocal photography

Myoblasts were cultured in the 35 mm cell culture dishes, after heat stress, the myoblasts were fixed at 4% paraformaldehyde at 4°C overnight. Then the myoblasts were permeabilized with 0.5% Triton X-100 for 20 min, and washed 3 times with phosphate buffer solution (PBS) (Solarbio, Beijing China) in the table concentrator, and blocked with 5% bovine serum albumin for 1 h, followed by incubation with the diluted antibodies: anti-PAX7, anti-MYF5 or anti-MYHC at 4°C overnight. Next, the myoblasts were washed with PBS in the table concentrator and incubated with the fluorescent secondary antibody for 1 h. Then the nuclei were incubated with DAPI (KeyGEN BioTECH, Jiangsu, China) for 15 min. After washing 3 times with PBS in the table concentrator, the cells were added with the anti-fluorescent quencher (Beyotime, Shanghai, China), then the laser scanning confocal microscopy (LSCM, Carl Zeiss, Oberkochen, Germany) was used to obtain the fluorescence images. ImageJ was used to compare the relative intensity of the cellular fluorescence between the CON and HS groups.

### 2.4 Western blot

The protein of the myoblasts was collected after heat stress using the radioimmune precipitation assay (RIPA) buffer (Beyotime, Shanghai, China) containing 1% phenylmethanesulfonylfluoride (PMSF) buffer (Beyotime, Shanghai, China) and the supernatant was collected after the centrifugation, then the concentration of protein was detected by a NanoDrop ND-2000 spectrophotometry (Thermo Fisher Scientific, Waltham, MA). Next, the SDS sample loading buffer (5×) (Solarbio, Beijing, China) was added to the supernatant and boiled at 95°C in the metal bath for 10 min to denature. 50 μg denatured protein was loaded to electrophorese in a 4%–20% SDS-PAGE gel to separate the protein, after the electrophoresis, the gel was transferred to a polyvinylidene fluoride (PVDF) membrane (absin, Shanghai, China) and blocked with 5% non-fat milk powder for 1.5 h at room temperature in the table concentrator. Then incubated with the primary antibody at 4°C overnight. GAPDH was the internal control. Then TBST (Solarbio, Beijing, China) was used to wash the membrane, after washing, the membrane was incubated with the corresponding horseradish peroxidase secondary antibody for 1.5 h. Finally, the band was exposed to the ECL chemiluminescence substrate (super sensitive) (Biosharp, Hefei, China) and performed with the Ultra-sensitive chemiluminescence gel imaging system (Tanon, Shanghai, China). The antibodies used in this study were listed in Supplementary Table S2. ImageJ (Wayne Rasband, MD, United States) was used to analyze the relative protein levels.

### 2.5 Transmission electron microscopy

The myoblasts were inoculated on the 6-well culture plates, after heat stress, the myoblasts were collected by trypsin for 5 min at 300 g, then PBS (Solarbio, Beijing, China) was used to wash the cells, and the myoblasts were fixed by the electron microscope fixative for 2 h. Then, the myoblasts were dehydrated by a series of ethanol (50%–70%–80%–90%–95%–100%) and acetone (100%), subsequently, the myoblasts were infiltrated by the compound of acetone and 812 embedding agents and polymerized in the oven at 60°C for 48 h. The ultrathin microtome (Leica, Wentzler, Germany) was used to cut the blocks into 60–80 nm-thick slides, then the slides were stained according to the study (Guo et al., 2019). Finally, the slides were observed by a transmission electron microscope (HITACHI, Tokyo, Japan) to collect images for further analysis.

### 2.6 Mitochondrial content measurement

The measurement of mitochondrial content was conducted following the previous study (Iwabu et al., 2010). mtDNA content and Mito-Tracker Green probe staining were used to evaluate the content of mitochondria. DNA of myoblasts was extracted using the TIANamp Genomic DNA Kit (Tiangen, Beijing, China), and the procedures were according to the instructions, then RT-qPCR was used to obtain the copy number of mtDNA using the relative ratio of mitochondrial COX2 to nuclear 18S rRNA, and the primer sequences were shown in Supplementary Table S1.

The myoblasts were cultured in the 35 mm cell culture dishes, and after heat stress, the cells were incubated with 100 nm Mito-Tracker Green (Beyotime, Shanghai, China) at 37°C for 40 min, then PBS was used to wash the cells to remove the Mito-Tracker Green. Finally, the fresh cell culture solution preheated at 37°C was added to observe the mitochondria using the laser scanning confocal microscope.

### 2.7 ROS measurement

The myoblasts were cultured in the 35 mm cell culture dishes, and after heat stress, the cells were incubated with 10 μM dichlorofluorescein diacetate (DCFH-DA) (Solarbio, Beijing, China) at 37°C for 20 min, after washing three times by DMEM/F12 to remove the extracellular DCFH-DA of myoblasts, the laser scanning confocal microscope was used to obtain the ROS images. ImageJ (Wayne Rasband, MD, United States) was used to detect the fluorescence levels of intracellular ROS.

### 2.8 Flow cytometry

The myoblasts were cultured in the 6-well culture plates, after heat stress, the cells were collected by 0.25% trypsin without ethylenediamine tetra acetic acid (EDTA) (Gibco, Grand Island, NY, United States), after washing two times by PBS, the cells were resuspended in the 500 μL binding buffer and incubated with 5 μL Annexin V-fluorescein isothiocyanate (FITC) (Thermo Pierce, Rockford, IL, United States) and 5 μL propidium iodide (PI) (Sigma-Aldrich, St. Louis, Missouri, United States). After a reaction of 10 min in the dark, the flow cytometer (Beckman Coulter, Brea, CA, United States) was used to detect the cells. FlowJo 10.8.1 was used to analyze the apoptosis cells.

### 2.9 Edu assay

The kFlouor555 Click-iT Edu Imaging Assay Kit (KeyGEN BioTECH, Jiangsu, China) was used to detect the proliferation of myoblasts. Specifically, the cells were incubated in the 35 mm cell culture dishes, after heat stress, the cells were incubated with 20 μM Edu working reagent for 2 h, then the Click-iT reaction mixture was added with a gentle shake to cover the cells, and incubated for 30 min at room temperature in the dark. Subsequently, 3% BSA in PBS was used to wash the cells to remove the reaction reagent. Then Hoechst33342 solution was added and incubated for 30 min at room temperature in the dark to stain the nucleus. Finally, the anti-fluorescence quenching agent was added and observed in the laser scanning confocal microscope.

### 2.10 Cell Counting Kit‐8 assay

The cells were seeded in the 96-well plates, and after heat stress, Cell Counting Kit-8 (CCK8) (ZETA LIFE, United States) was added to the growth medium, and changed the original growth medium of the cells, after incubation at 37°C for 2 h, the spectrophotometer (Thermo Fisher Scientific, Waltham, MA) was used to detect the absorbance of the cells at 450 nm.

### 2.11 Detection of mitochondrial membrane potential (Δψm)

The Δψm was determined by the Mitochondrial Membrane Potential Assay Kit (JC-1, abs50016, absin, Shanghai, China). Specifically, the cells were incubated in the 35 mm culture dishes, after heat stress, the original cell culture medium was removed, and 1 mL new cell culture medium and 1 mL JC-1 working solution were added and incubated at 37°C for 20 min. Then, the cells were rinsed twice with JC-1 buffer (1×), after washing, 2 mL cell culture medium was added. Finally, the laser scanning confocal microscope was used to detect the fluorescence levels of the JC-1 polymers and monomers. ImageJ was used to analyze the relative ratio of the red fluorescence to the green fluorescence.

### 2.12 Wound-healing assay

The cell migration of the myoblasts was determined by the wound-healing assay. Briefly, the cells were seeded in the 6-well culture plates, after heat stress, a sterile 1 mL pipette tip was used to smear with a regular wound, then the inverted microscope (Nikon, Japan) was used to monitor the images after 0, 24, 48, and 72 h. ImageJ was used to detect the wound healing area.

### 2.13 Statistical analysis

All experiments were repeated three times. The data of the statistical analysis was conducted by SPSS 23.0 (SPSS Inc., Chicago, IL, United States) and presented as the mean ± SEM. One-way analysis of variance (ANOVA) was used to compare the differential significance between the two groups. The significant difference was shown as *p* < 0.05 (*), *p* < 0.01 (**) and *p* < 0.001 (***), respectively.

## 3 Results

### 3.1 Identification of heat stress model of myoblasts *in Vitro*

**FIGURE 1:**
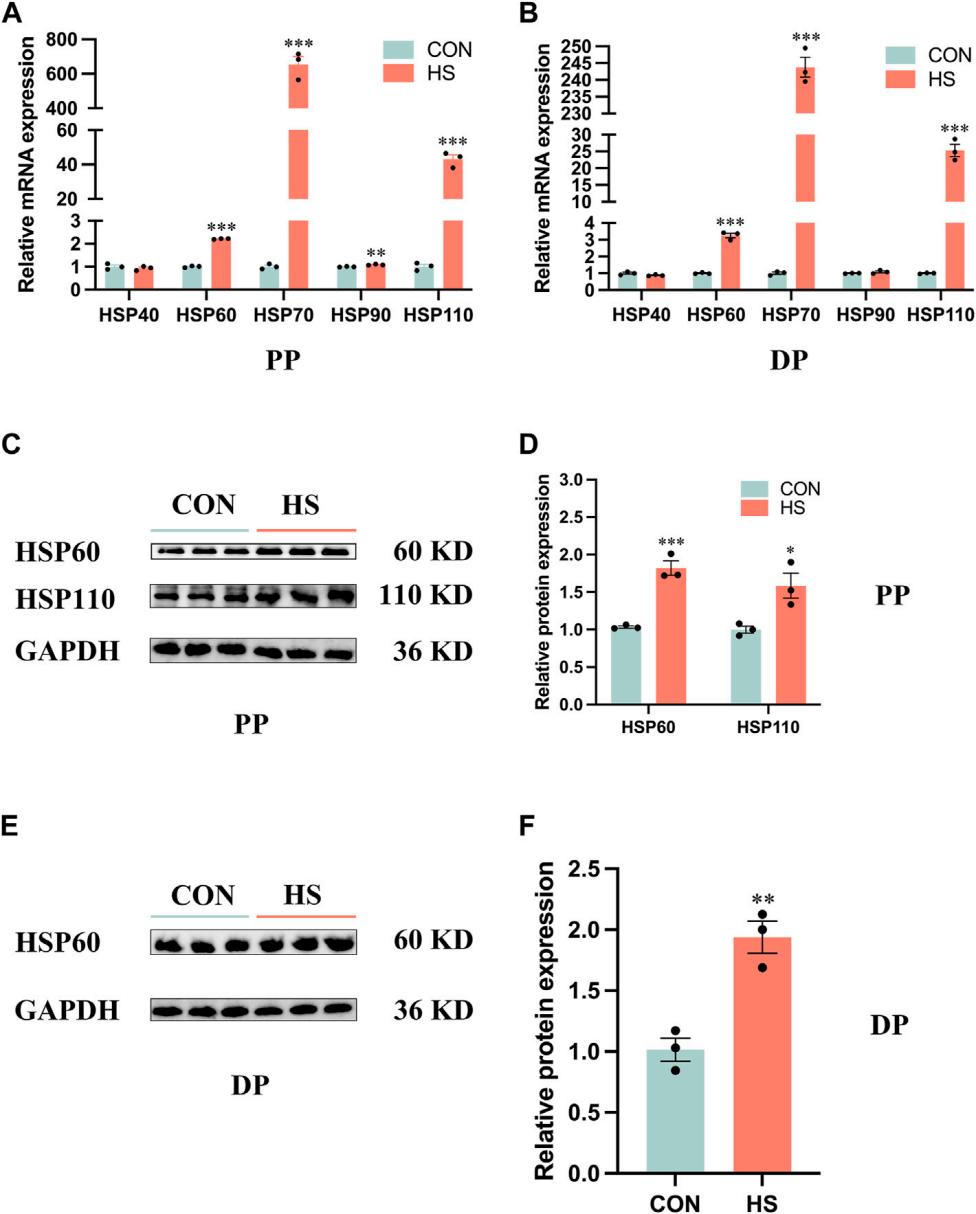
Heat stress upregulated the expression levels of the HSP family in myoblasts. mRNA expression of the HSP family in myoblasts during proliferation **(A)** and differentiation **(B)**. Protein levels of the HSP family in myoblasts during proliferation **(C, D)** and differentiation **(E, F)**. All data are shown as the mean ± SEM (n = 3, *p < 0.05; **p < 0.01, ***p < 0.01). PP: proliferation phase; DP: differentiation phase.

To verify the myoblasts were in a heat-stressed condition, we detected the expression of HSP families of protein and mRNA levels (Figure 1). RT-qPCR results indicated the mRNA expression of *HSP90* (*p* < 0.01), *HSP60*, *HSP70* and *HSP110* (*p* < 0.001) of myoblasts during proliferation was significantly increased (Figure 1A). Similarly, heat stress significantly increased the mRNA expression of *HSP60*, *HSP70* and *HSP110* (*p* < 0.001) of myoblasts during differentiation by RT-qPCR analysis (Figure 1B). Western blot results indicated that the protein levels of HSP60 (*p* < 0.001) and HSP110 (*p* < 0.05) were significantly increased in the heat-stressed myoblasts during proliferation (Figures 1C, D), a significant increase of HSP60 protein level was also detected in the heat-stressed myoblasts during differentiation (Figures 1E, F). Overall, these results indicated that we performed a heat-stressed model of myoblasts *in vitro*.

### 3.2 Heat stress inhibited the proliferation of myoblasts

**FIGURE 2:**
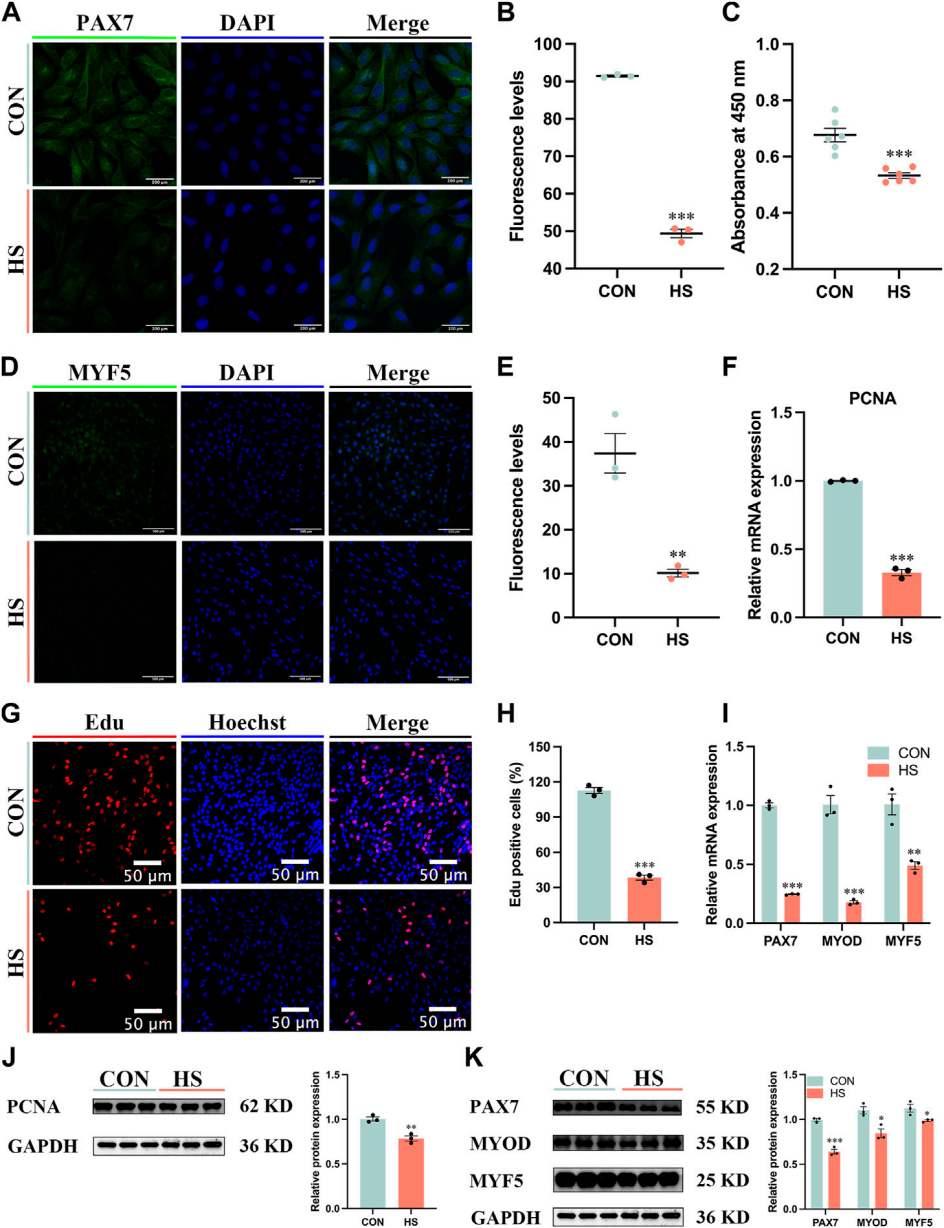
Heat stress inhibited the proliferation of myoblasts. Representative images of **(A)** PAX7, Scale bar, 200 μm, and **(D)** MYF5 by immunofluorescence in myoblasts at proliferation. Scale bar, 500 μm. The fluorescence levels of PAX7 **(B)** and MYF5 **(E)** in myoblasts at proliferation. **(C)** The cell viability of myoblasts was detected by CCK8. mRNA **(F)** and protein **(J)** expression of PCNA in myoblasts at proliferation. **(G)** Edu assay detected the cell proliferation of myoblasts. Scale bar, 50 μm. **(H)** The Edu-positive cells of myoblasts. mRNA **(I)** and protein **(K)** expression of PAX7, MYOD and MYF5 in myoblasts at proliferation. All data are shown as the mean ± SEM (n = 3, *p < 0.05; **p < 0.01, ***p < 0.01).

To explore the effects of heat stress on the proliferation of myoblasts, the immunofluorescence assay was conducted to observe the expression of myoblast markers during proliferation (Figures 2A–D). We discovered heat stress significantly attenuated the fluorescence levels of PAX7 (*p* < 0.001, Figure 2B) and MYF5 (*p* < 0.01, Figure 2E). The cell viability of heat-stressed myoblasts was significantly reduced by CCK8 assay (*p* < 0.001, Figure 2C). The mRNA and protein expression of cell proliferation marker PCNA was significantly decreased (*p* < 0.01) in heat-stressed myoblasts compared to CON myoblasts (Figures 2E–J). The Edu assay showed that the Edu-positive cells were significantly decreased (*p* < 0.001) in the HS group compared to the CON group (Figures 2G, H). Additionally, the mRNA expression of *MYF5* (*p* < 0.01), *PAX7* and *MYOD* (*p* < 0.001) was significantly lower in the HS group than in the CON group by RT-qPCR (Figure 2I). Likewise, the protein levels of PAX7 (*p* < 0.001), MYOD and MYF5 (*p* < 0.05) were significantly decreased in the heat-stressed myoblasts by Western blot (Figure 2K). Taken together, heat stress had an adverse impact on the proliferation of myoblasts.

### 3.3 Heat stress inhibited myoblast myogenic differentiation

**FIGURE 3:**
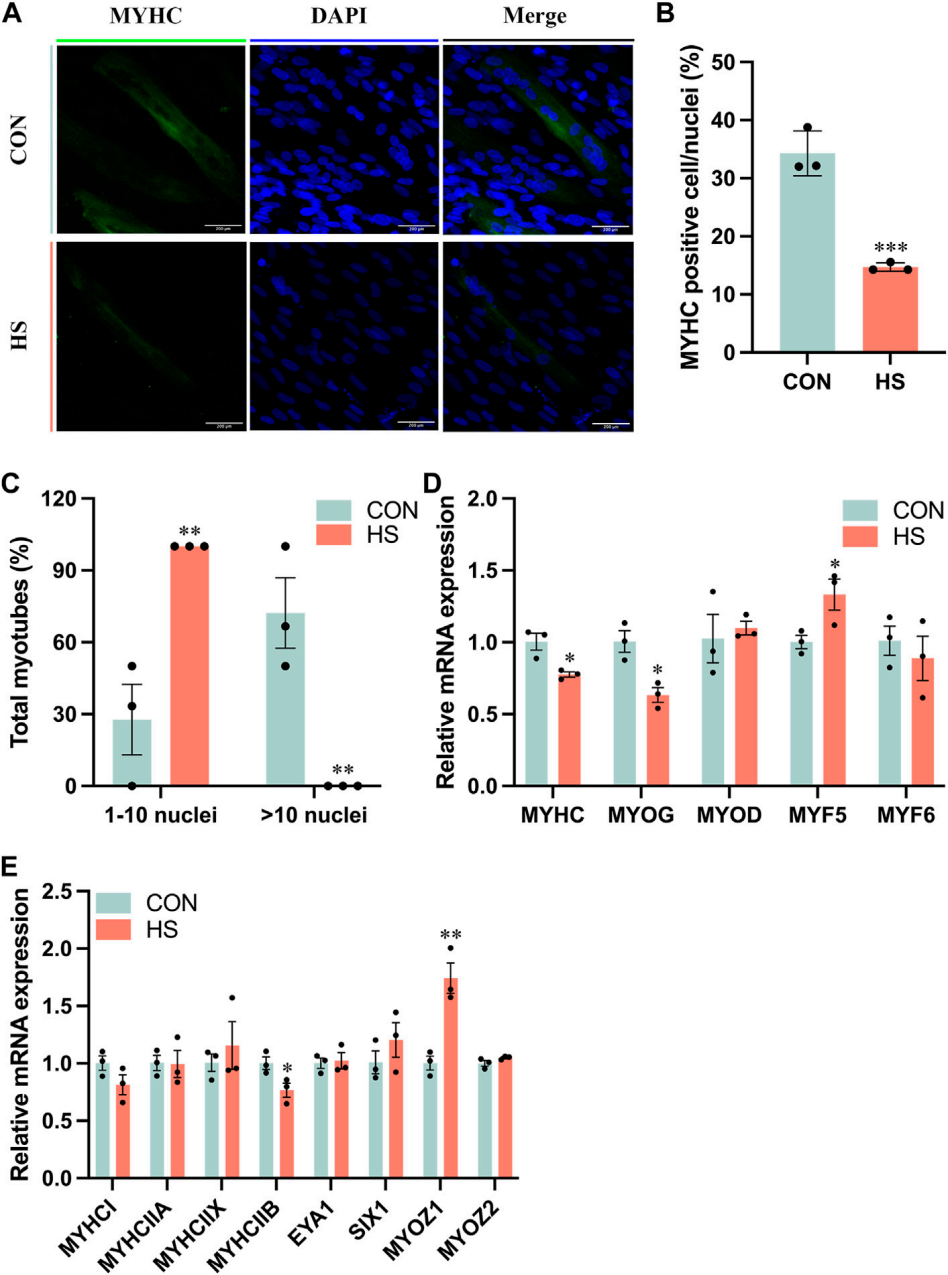
Heat stress inhibited the differentiation of myoblasts. **(A)** Representative images of MYHC by immunofluorescence in myoblasts during differentiation. Scale bar, 200 μm. **(B)** The MYHC-positive cells of myoblasts. **(C)** The total myotubes of myoblasts. **(D)** RT-qPCR detected the expression of the differential genes. **(E)** RT-qPCR detected the expression of the genes related to the muscle fiber type. All data are shown as the mean ± SEM (n = 3, *p < 0.05; **p < 0.01, ***p < 0.01).

To investigate the impact of heat stress on myogenic differentiation, we performed the immunofluorescence assay of MYHC to estimate the formation of myoblasts (Figure 3A), the staining results indicated that MYHC^+^ cells were significantly decreased in heat-stressed myoblasts compared to the CON (*p* < 0.001, Figure 3B), accompanied with a reduction of myotube sizes (*p* < 0.01, Figure 3C). Meanwhile, the mRNA levels of *MYHC* and *MYOG*, the markers of myogenic differentiation, were significantly reduced, and *MYF5* (*p* < 0.05) was significantly increased in myoblasts under heat stress compared to the CON myoblasts (Figure 3D). mRNA level of *MYHCIIB* was significantly decreased (*p* < 0.05) and *MYOZ1* was significantly enriched (*p* < 0.01) of myoblasts after heat stress by RT-qPCR (Figure 3E). From the above results, heat stress inhibited myogenic differentiation and reduced the fast fiber of myoblasts.

### 3.4 Heat stress inhibited myoblast migration

**FIGURE 4:**
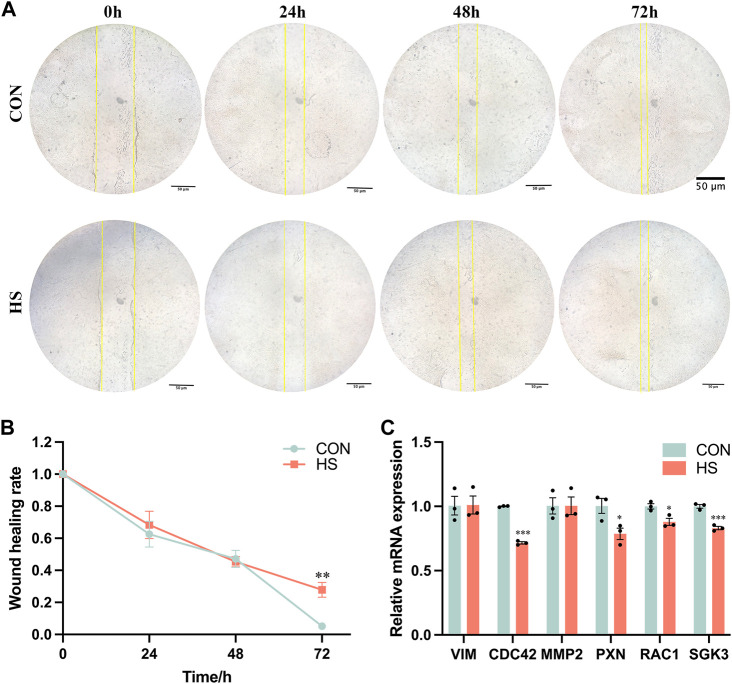
Heat stress inhibited the cell migration of myoblasts. **(A)** Representative images of the myoblasts by a wound-healing assay. **(B)** The wound healing rate of the myoblasts. **(C)** mRNA expression of the migration-related genes in myoblasts by RT-qPCR. All data are shown as the mean ± SEM (n = 3, *p < 0.05; **p < 0.01, ***p < 0.01).

We performed the wound-healing assay to detect the migratory ability of myoblasts under heat stress (Figure 4A), and the results indicated that heat stress impeded the migratory ability of myoblasts after 72 h (*p* < 0.01, Figure 4B). The mRNA levels of genes related to cell migration, including *CDC42* and *SGK3* (*p* < 0.001), *PXN* and *RAC1* (*p* < 0.05) were pronouncedly reduced, *VIM* and *MMP2* were not significant in myoblasts under heat stress (*p* > 0.05, Figure 4C). Overall, these results demonstrated that heat stress inhibited the migration of myoblasts.

### 3.5 Heat stress promoted autophagy and apoptosis of myoblasts

**FIGURE 5:**
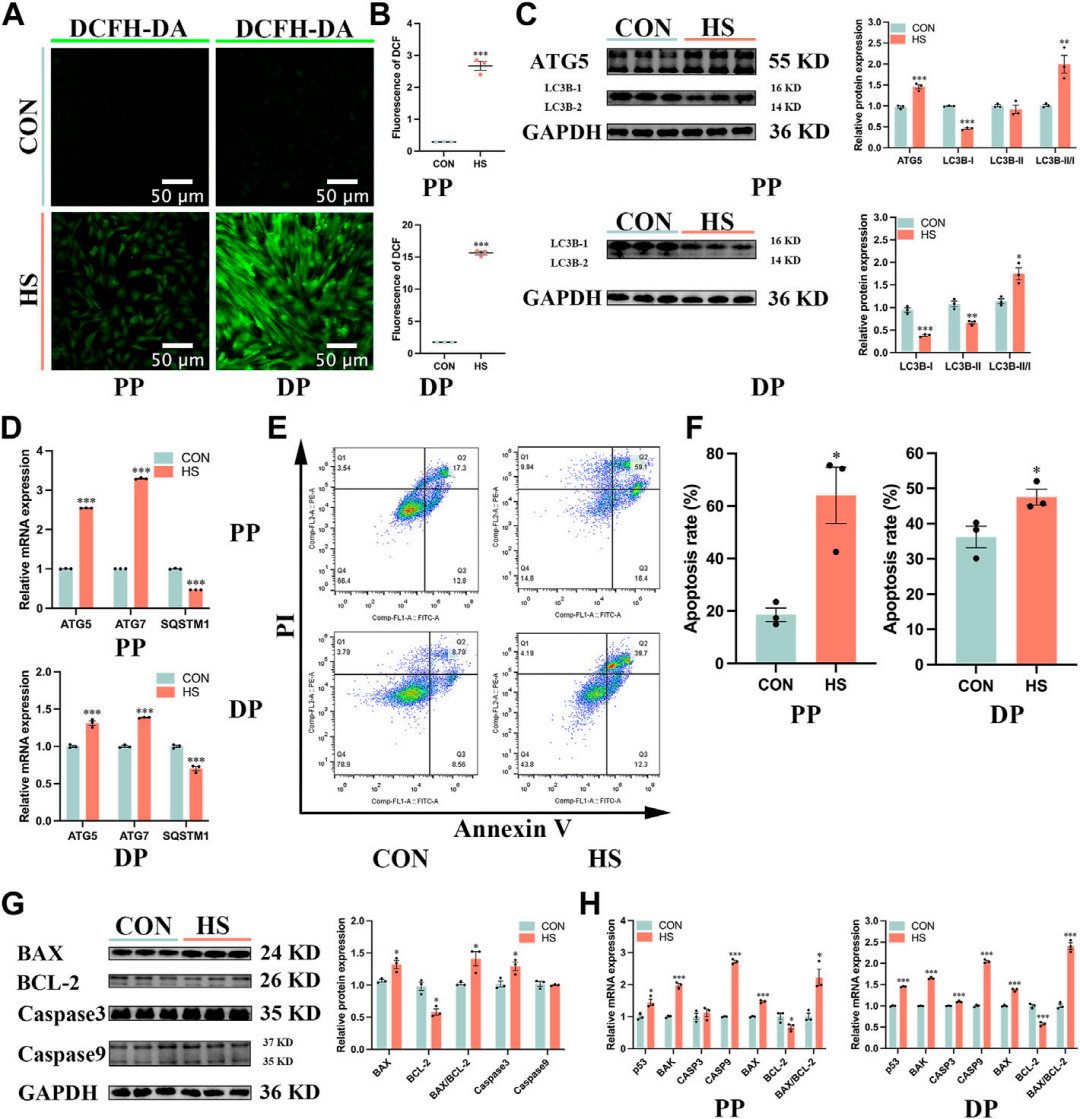
Heat stress accelerated the autophagy and apoptosis of myoblasts. **(A)** The fluorescent images of myoblasts stained by DCFH-DA probe during proliferation and differentiation. Scale bar, 50 μm. **(B)** The fluorescence levels of DCFH-DA. **(C)** Protein expression of autophagy in myoblasts during proliferation and differentiation. **(D)** mRNA expression of autophagy in myoblasts during proliferation and differentiation. **(E)** The apoptosis of myoblasts during proliferation and differentiation was determined by flow cytometry. **(F)** The apoptosis rate of myoblasts was detected by flow cytometry. **(G)** Protein expression of apoptosis in myoblasts during proliferation. **(H)** mRNA expression of apoptosis in myoblasts during proliferation and differentiation. All data are shown as the mean ± SEM (n = 3, *p < 0.05; **p < 0.01, ***p < 0.01).

To investigate the effects of heat stress on the apoptosis of myoblasts, we first detected the levels of cellular ROS by DCFH-DA probe (Figure 5A), the results showed that ROS levels of myoblasts under heat stress were significantly enriched during proliferation and differentiation (*p* < 0.001, Figure 5B). Then we examined the effects of heat stress on the autophagy of myoblasts. The protein levels of ATG5 (*p* < 0.001) and LC3B-II/I (*p* < 0.01) were significantly increased, the protein levels of LC3B-I (*p* < 0.001) and LC3B-II (*p* < 0.05) were significantly reduced in the heat-stressed myoblasts during proliferation; the protein levels of LC3B-I (*p* < 0.001) and LC3B-II (*p* < 0.01) were significantly decreased, and the ratio of LC3B-II/I (*p* < 0.05) were significantly increased in heat-stressed myoblasts during differentiation (Figure 5C). Besides, the mRNA levels of *ATG5* and *ATG7* were significantly reduced and *SQSTM1* was significantly increased in the heat-stressed myoblasts during proliferation and differentiation (*p* < 0.001, Figure 5D). And we found heat stress significantly increased the apoptosis cells during proliferation and differentiation by the flow cytometry analysis (*p* < 0.05, Figures 5E, F). Additionally, heat stress significantly reduced the protein levels of BCL-2 (*p* < 0.05), and significantly increased the protein levels of BAX, BAX/BCL-2 and Caspase3 of myoblasts during proliferation (*p* < 0.05, Figure 5G). RT-qPCR results indicated that the mRNA levels of *p53*, *BAK*, *Caspase9* and *BAX/BCL-2* in heat-stressed myoblasts were significantly increased, and the mRNA levels of *BCL-2* were significantly reduced during proliferation and differentiation (*p* < 0.05, Figure 5H). Taken together, these results indicated that heat stress promoted the autophagy and apoptosis of myoblasts.

### 3.6 Heat stress impaired myoblast mitochondrial biogenesis and function

**FIGURE 6:**
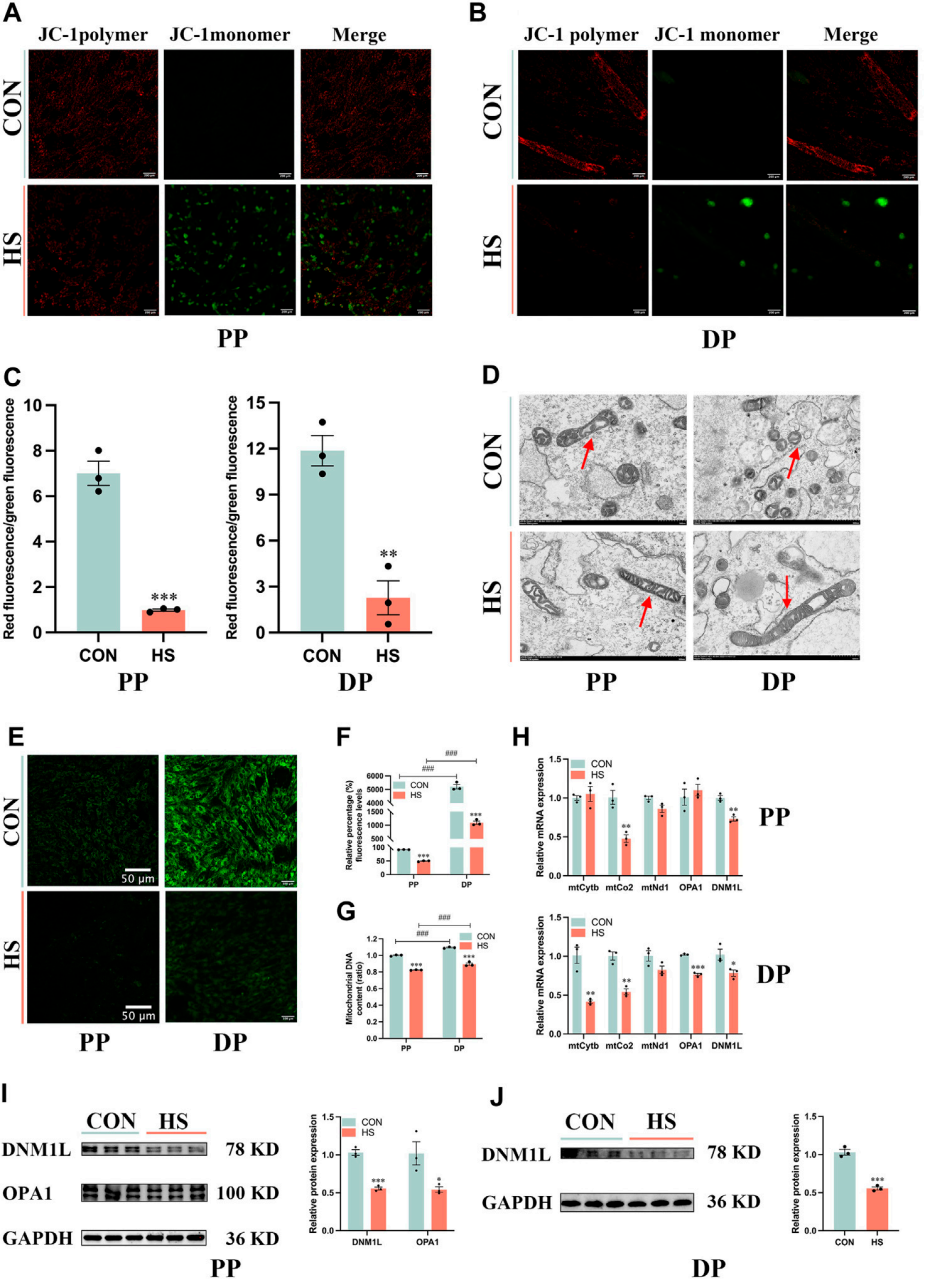
Heat stress impaired the mitochondrial biogenesis and function of myoblasts. Representative images of myoblasts stained by JC-1 during proliferation **(A)** and differentiation **(B)**. **(C)** The ratio of the red fluorescence to the green fluorescence. **(D)** Electron micrographs of myoblasts during proliferation and differentiation. Red arrowheads indicate mitochondria. Scale bar, 500 nm. **(E)** Representative images of myoblasts stained by Mito-Tracker Green probe during proliferation and differentiation. **(F)** The relative percentage of Mito-Tracker Green fluorescence levels. **(G)** Mitochondrial DNA content of myoblasts at proliferation and differentiation. **(H)** mRNA expression of mitochondrial DNA-encoded genes of myoblasts at proliferation and differentiation. Western blot analysis of the expression of DNM1L and OPA1 in myoblasts at proliferation **(I)** and differentiation **(J)**. All data are shown as the mean ± SEM (n = 3, *p < 0.05; **p < 0.01, ***p < 0.01, and ###*p* < 0.001 compared with PP.

To illustrate the effects of heat stress on mitochondrial biogenesis and function in myoblasts, we detected Δψm of myoblasts during proliferation and differentiation (Figures 6A, B). Heat-stressed myoblasts exhibited lower Δψm during proliferation (*p* < 0.001) and differentiation (*p* < 0.01, Figure 6C). Transmission electron microscopy was used to observe the size and morphology of mitochondria in myoblasts under heat stress. The electron micrographs exhibited that most mitochondria presented spherical and only a few mitochondria were elongated in myoblasts, and heat stress diminished the number of mitochondria in myoblasts during proliferation and differentiation (Figure 6D). The mitochondrial morphology of myoblasts was measured *via* the Mito-tracker green probe, which indicated that differentiated myoblasts had more mitochondria than undifferentiated myoblasts (*p* < 0.001), and heat stress impaired mitochondrial biogenesis during proliferation and differentiation (*p* < 0.001, Figures 6E, F). Moreover, differentiated myoblasts had higher mitochondrial DNA content than undifferentiated myoblasts (*p* < 0.001), and heat stress significantly decreased the mitochondrial DNA content of myoblasts during proliferation and differentiation (*p* < 0.001, Figure 6G). Besides, under heat stress, the mRNA levels of *mtCo2* and *DNM1L* were significantly decreased during proliferation (*p* < 0.01, Figure 6H), the mRNA levels of *mtCytb*, *mtCo2*, *OPA1* and *DNM1L* were significantly reduced during differentiation (*p* < 0.05, Figure 6H). Additionally, heat stress led to the downregulation of the protein levels of DNM1L (*p* < 0.01) and OPA1 (*p* < 0.05) during proliferation (Figure 6I) and DNM1L (*p* < 0.001) of myoblasts during differentiation (Figure 6J). These results suggested that the mitochondria of heat-stressed myoblasts were impaired and lost function during proliferation and differentiation.

## 4 Discussion

**FIGURE 7:**
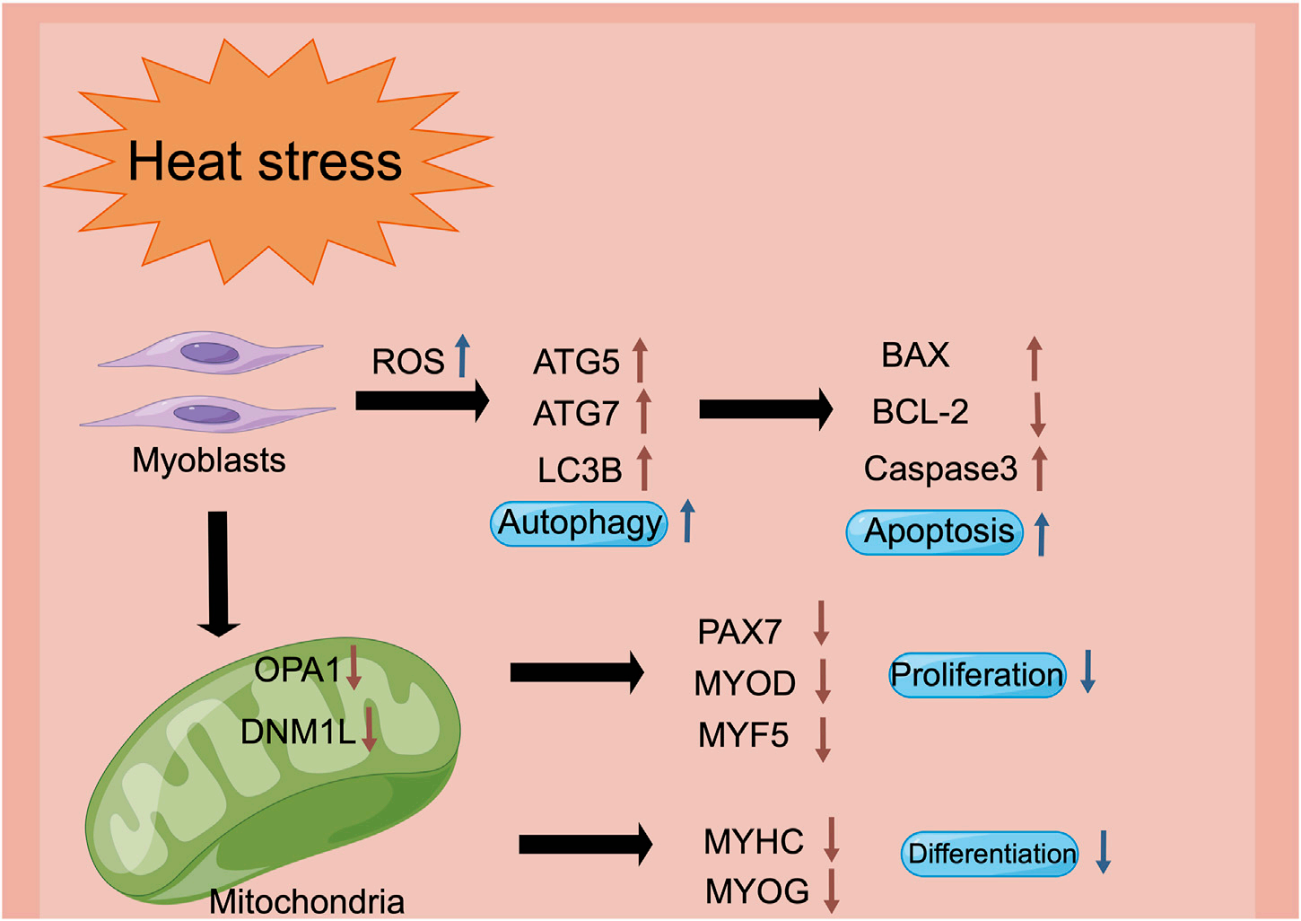
The schematic diagram of heat stress modulates the biological processes of myoblasts *via* the regulation of the mitochondrial function (figure made by Figdraw). Heat stress impairs mitochondrial biogenesis and function, stimulation of the intracellular reactive oxygen species, and decrease of the mitochondrial membrane potential, which may be the reason for the inhibition of proliferation, differentiation and cell migration in the myoblasts.

Heat stress is one of the vital environmental factors that reduces the muscle quality of livestock and poultry by regulating the physiological and metabolic mechanisms. Heat stress facilitated autophagy, and induced the apoptosis of the myoblasts. Besides, heat stress restrained mitochondrial biogenesis and function, which led to the inhibition of the proliferation, differentiation and migration of the myoblasts, thereby affecting the growth and development of muscle (Figure 7). These findings could provide a potential mechanism reference for the damage of heat stress on the growth and development of human muscles.

Heat stress directly changes the activity of the satellite cells to regulate the growth of skeletal muscles (Loyau et al., 2013; Piestun et al., 2013). Heat shock response is activated when the cells or tissues are in a heat-stressed condition (Velichko et al., 2013). This study indicated that HSP60 was mainly located in the cytoplasm, HSP70 and HSP110 were expressed in both the nucleus and cytoplasm of Hu sheep myoblasts during differentiation, which is in agreement with a previous report (Thakur et al., 2019). The cells immediately generate heat shock proteins (HSPs) to prevent the pernicious impact of heat stress (Al-Aqil and Zulkifli, 2009). HSPs produce a remarked effect on modulating cellular homeostasis as chaperones (Hartl and Martin, 1996). HSP families include HSP40 (35–54 kDa), HSP60 (55–65 kDa), HSP70 (65–80 kDa), HSP90 (81–99 kDa) and HSP110 (≥100 kDa) according to the molecular weights (Thakur et al., 2019). HSP60 of the cytoplasm inhibits caspase3 to protect the cells from apoptosis (Lin et al., 2001; Kirchhoff et al., 2002; Zhang et al., 2004). HSP60 is involved in the processes of protein folding and mitochondrial biogenesis (Deocaris et al., 2006). HSP70 is the most conservative and abundant endogenous protective protein in the HSPs family, which protects the denatured protein folding, unfolding and refolding as molecular chaperones (Nollen et al., 1999). HSP70 is the most sensitive member of the HSPs family to temperature changes (Yang et al., 2007). Some studies have reported that the increased level of HSP70 in the organisms is an important sign of animals undergoing heat stress (Nagayach RichiD-Gupta and Prakash, 2017; Hu et al., 2019), which proves that we have successfully established a heat-stressed model *in vitro*. HSP110 could recognize and maintain the solubility and refold ability of the denatured protein as a high molecular chaperone (Oh et al., 1997). We observed the increased expression levels of HSP60, HSP70 and HSP110 in the heat-stressed myoblasts during proliferation and differentiation, which indicated that the myoblasts suffered from severe heat stress. Metzger et al. (2022) reported that heat stress (41°C) upregulated the expression of HSP70 in porcine myoblasts (Metzger et al., 2022).

The development of skeletal muscle is an extraordinarily complicated and delicate process, which includes the proliferation and differentiation of the satellite cells (Reed et al., 2022). Myoblasts proliferate into muscle cells, and then fuse with each other to form the myotubes, which gradually converge to form the muscle fibers, finally, the skeletal muscle is formed (Romagnoli et al., 2021). Myoblast is the key component of muscle movement and regeneration, which proliferates and differentiates to form new muscle fibers and maintain skeletal muscle health (Bolus et al., 2018). Therefore, myoblasts are selected as the research object in this study to explore heat stress on the proliferation and differentiation of skeletal muscle *in vitro*. Mitochondria exert a functional impact on regulating the proliferation and differentiation of myoblasts (Remels et al., 2010; Sin et al., 2016). Paired box 7 (PAX7) is a vital upstream regulator in the formation of skeletal muscle and maintains the pluripotency of the satellite cells, which makes a difference in modulating the growth and regeneration of skeletal muscle (Buckingham and Relaix, 2015). Myogenic differentiation (MYOD) and myogenic factor 5 (MYF5) accelerate the proliferation of the myoblasts, myogenin (MYOG) leads myoblasts to differentiate into muscle cells, and together with MYOD and MYF5, regulates the fusion of muscle cells into multinucleated muscle fibers (Rudnicki et al., 1993; Andres and Walsh, 1996; Meadows et al., 2008). Heat stress impaired mitochondrial biogenesis and function, thereby reducing the expression of PAX7, MYOD, MYF5, MYOG and MYHC, which might be the reason for the inhibition of proliferation and differentiation in myoblasts. Metzger et al. (2022) demonstrated that the biological processes and signaling pathways correlated with proliferation were downregulated in porcine myoblasts under heat stress (41°C) (Metzger et al., 2022), and Chen et al. (2020) reported that heat stress (42°C) interfered the myogenic differentiation *via* changing the expression of MYOD, MYOG and MYHC (Chen et al., 2020), which is consistent with our results.

Cell migration, proliferation and differentiation have profound effects on the development of skeletal muscle (Ye et al., 2020). SGK3 pertains to the SGK family and regulates a lot of biological processes, which accelerates cell migration and invasion in hepatocellular cells (Kong et al., 2016; Ye et al., 2020). Cell division cycle 42 (CDC42) could improve cell migration *via* the PTEN/AKT pathway (Huang et al., 2022). Paxillin (PXN) enriches cell migration and angiogenesis as adhesive molecules (Yang et al., 2020). Racfamily small GTPase 1 (RAC1) may promote the migration of MCF-7 breast cancer cells (Tian et al., 2018). In our study, heat stress enriched the wound healing rate of myoblasts after 72 h, and downregulated the expression of CDC42, PXN, RAC1 and SGK3, leading to the inhibition of the myoblasts migration.

ROS is a highly active molecule derived from oxygen that maintains the stability of the organism and partakes in the signal transduction of cell metabolism (Postiglione and Muday, 2020; Liu et al., 2022). Exogenous stimulation facilitates the production of cellular ROS (Kasai et al., 2020). Heat stress enriches the content of cellular ROS and induces the apoptosis of the myoblasts in this study, excessive ROS breaks the cell constituent, such as nucleic acids and proteins, which causes cell death (Imlay, 2013). Autophagy is activated to eliminate the harmful effect of ROS to maintain intracellular homeostasis (Palmeira et al., 2019). Activation of autophagy is triggered under the condition of increased ROS, and ROS is considered necessary to induce autophagy of cells (Lin et al., 2019), which might be the reason that autophagy is enriched in the heat-stressed myoblasts. Autophagy is the preponderant intracellular degradative system to maintain the homeostasis of cells as a circulation system, which plays a key role in restoring the normal physiological metabolism of cells under pressure (Mizushima and Komatsu, 2011; Ma et al., 2020). Myoblasts alleviate the negative influence of heat stress *via* autophagy to renew intracellular homeostasis.

The mitochondrion is the source of cellular endogenous ROS, and ROS is a trigger factor of mitochondrial dysfunction (Li et al., 2020). The abnormal accumulation of ROS results in the reduction of mitochondrial membrane potential (Peoples et al., 2019). Additionally, mitochondria have their own genome, mitochondrial DNA (mtDNA), and defects of mtDNA promote the generation of ROS (Annesley and Fisher, 2019; Palmeira et al., 2019). We observed the reduction of mitochondrial membrane potential and mtDNA in the heat-stressed myoblasts, which is consistent with a previous study, heat stress (43°C) enriched the excessive ROS and apoptosis, reduced the cell viability and mitochondrial membrane potential in C2C12 myoblasts (Yu et al., 2022). Mitochondria are the major organelles to produce adenosine triphosphate (ATP) in eukaryotic cells (Van Der Bliek et al., 2017; Annesley and Fisher, 2019). In our study, we found heat stress reduced the number of mitochondria, while decreasing the expression of mtCo2, DNM1L and OPA1 of myoblasts during proliferation and differentiation, indicating that mitochondrial biogenesis and function were impaired in the heat-stressed myoblasts.

Conclusively, we found that heat stress promoted the apoptosis of the myoblasts by accelerating autophagy, besides, heat stress had an adverse impact on the proliferation and differentiation of the myoblasts, and the possible mechanism of these may be the damage to the mitochondria, leading to the inhibition of the cell migration. Our results elucidated that heat stress inhibited the biological processes of the myoblasts, thereby providing the basis for heat stress that impairs the development of the skeletal muscle.

## Data Availability

The raw data supporting the conclusions of this article will be made available by the authors, without undue reservation.
