# Supplementary material for: Heat stress inhibits the proliferation and differentiation of myoblasts and is associated with damage to mitochondria
Source: Front Cell Dev Biol. 2023 Apr 11;11:1171506. doi: 10.3389/fcell.2023.1171506 (PMC10126414; doi:10.3389/fcell.2023.1171506)
Supplement: Supplementary file 1 [file Table1.docx]

Supplementary Material

Heat stress inhibits the proliferation and differentiation of myoblasts and is associated with damage to mitochondria

Jiawei Lu, Huixia Li*, Debing Yu*, Peng Zhao, Yuan Liu

*** Correspondence:** Huixia Li: lihuixia@njau.edu.cn; Debing Yu: yudebing@njau.edu.cn

# Supplementary Table 1

The primer sequences of genes for RT-qPCR.

| **Gene** | **Forward Primer (5’-3’)** | **Reverse Primer (5’-3’)** |
| --- | --- | --- |
| *GAPDH* | AGGATCTCGCTCCTGGAAGAT | CCGTTCGACAGATAGCCGTAA |
| *HSP40* | GGTGGCTATTACAGAGGTGGGTTTG | TGGCTTTATTGGCAGTAGGGCTTAC |
| *HSP60* | GTCCAGTCCATTGTTCCTGCTCTTG | TTGACCGCCACAACTTGAAGACC |
| *HSP70* | CGACGACGGCATCTTCAAGGTG | TGTTCTGGCTGATGTCCTTCTTGTG |
| *HSP90* | GTGGACACGCTCAGCAAGGATG | CACCTCTTCCGACTCCTCCTCTG |
| *HSP110* | TGCCTATTGAAGCCAACTTGGTCTG | CATACTCCTCCACCGCATTCTTAGC |
| *ATG5* | TGTGCTTCGAGATGTGTGGTTTGG | TGGTTCTGCCTCACGTTCAGTTATC |
| *ATG7* | AAGGTTGTGTCTGCCAAGTGTCTG | TTGTCCACGAACGTGATGTGTCTG |
| *SQSTM1* | TCAGCCTGAGGAACAGATGGAGTC | AGAGACTGGAGTTCACCTGTAGACG |
| *p53* | TCAGATCCGTGGGCGTAAAC | AGTCTGAGTCAGGCCCTTCT |
| *BAK* | CCCCATCAAGAACGTAGCCA | GTCTCAGGACCTCAAAGGGC |
| *BAX* | GCATCCACCAAGAAGCTGAG | CCGCCACTCGGAAAAAGAC |
| *BCL-2* | ATGTGTGTGGAGAGCGTCA | AGAGACAGCCAGGAGAAATC |
| *Caspase3* | TGAAACATGCCGCCTTCCTA | AGTGGCATACCCACATGACTG |
| *Caspase9* | CCAGATGCCGTGTCTAGTCTG | ACAGTAAGGTAGGGTGAGGGG |
| *18S rRNA* | GTGATGGGGATCGGGGATTG | GTAGCGACGGGCGGTGTGTA |
| *COX2* | CAGGATCGAACAACCCCACA | TGTCTGGGTCTCCGAGTAGG |
| *mtCytb* | TTGGGCGGAATATTGGGCTT | TGCATACGCCATTCTACGCT |
| *mtCo2* | ACAGACGCAATTCCAGGTCG | TGGGACTAGCTCGAGAACGA |
| *mtNd1* | ATGGGCTGTGAAATGCTCCT | CCTGACCGAAGGAGAATCCG |
| *OPA1* | GCGAGGTCTGCCAATCCTTAGTG | CGTCCTTATCTGGGGTCTTTGAAGC |
| *DNM1L* | ACTTGTGGACTTGCCAGGAATGAC | CAGCAGTGACAGCGAGGATAATGG |
| *PCNA* | GTCCAGGGCTCCATCTTGAAGAAAG | GCTCTGTAGGTTCACGCCACTTG |
| *PAX7* | GTGCCCTCAGTGAGTTCGAT | CTGCTTACGCTTCAGAGGGA |
| *MYOD* | GCACGTCTAGCAACCCAAAC | AGTAAGCACGGTCGTAGCAG |
| *MYF5* | TGAAGAAGGTCAACCAGGCTTTCG | GCAGGCTCTCAATGTAGCGGATG |
| *MYHC* | CTGTCCAAGTTCCGCAAGGT | GAGCTTCGTTGCACCCTCAA |
| *MYOG* | AGAACTACCTGCCTGTCCACCTC | CCGACTTCCTCTTGCACACCTTAC |
| *MYF6* | GCTCGTGATAACTGCCAAGGAAGG | CCACGCAGGGGAGTTTGTGTTC |
| *MYHCI* | ACCAACCTGTCCAAGTTCCG | CGCGGCTACTCCTCATTCAA |
| *MYHCIIA* | AAGGGCTGACATTGCTGAGT | TGCCTCTCTTCAGTCATTCCA |
| *MYHCIIX* | GGTCTACGCAAACACGAGAG | GCGGAATTTGGAGAGGTTGAC |
| *MYHCIIB* | GACAACTCCTCTCGCTTTGG | GGACTGTGATCTCCCCTTGA |
| *EYA1* | CCGTCCACGAATGCCACTTACC | GGCTATGGATTGTACCGTACTCTGC |
| *SIX1* | CATCGTTCGGCTTCACACAGGAG | GCCTTGAGCACGCTTTCATTCTTG |
| *MYOZ1* | GGATTCTCCTACAGCAAGAGCAGTG | CAGAGCCCTGACGATGGTGTTG |
| *MYOZ2* | AAGCAGAGGAAAGAGCAAGCATCAG | CTGGCACCACGGTTACTGAGATG |
| *VIM* | TCAATGACCGCTTCGCCAACTAC | GCTCTCGCATCTCCTCCTCGTAG |
| *CDC42* | GCTGTCAAGTATGTGGAGTGCTCTG | CTGCGGCTCTTCTTCGGTTCTG |
| *MMP2* | GATAACCTGGATGCTGTGGTGGAC | TGCTTCCGAACTTCACGCTCTTC |
| *PXN* | TGAACGCCGTGCAGCATAACC | TATTCTCTGGGACGCCGTAGTGG |
| *RAC1* | CACTGTCCCAACACACCCATCATC | GGCGTCAGCTTCTTCTCCTTCAG |
| *SGK3* | GGCAGGAGTGAGTGGTTTGTCTTC | GCAGGAATCTTCAGGGCCATAGC |

# Supplementary Table 2.

The details of the antibodies used for WB and IF in this study.

| **Antibodies** | **Cat No.** | **Source** | **Dilution of WB** | **Dilution of IF** |
| --- | --- | --- | --- | --- |
| GAPDH | 60004-1-IG | Proteintech, Wuhan, China | 1:8000 |  |
| HSP60 | 15282-1-AP | Proteintech, Wuhan, China | 1:2000 |  |
| HSP110 | 13383-1-AP | Proteintech, Wuhan, China | 1:1000 |  |
| ATG5 | 10181-2-AP | Proteintech, Wuhan, China | 1:1000 |  |
| LC3B | 14600-1-AP | Proteintech, Wuhan, China | 1:1000 |  |
| BAX | 50599-2-Ig | Proteintech, Wuhan, China | 1:5000 |  |
| BCL-2 | 12789-1-AP | Proteintech, Wuhan, China | 1:1000 |  |
| Caspase3 | 19677-1-AP | Proteintech, Wuhan, China | 1:1000 |  |
| Caspase9 | 10380-1-AP | Proteintech, Wuhan, China | 1:500 |  |
| DNM1L | 12957-1-AP | Proteintech, Wuhan, China | 1:2000 |  |
| OPA1 | 27733-1-AP | Proteintech, Wuhan, China | 1:2000 |  |
| MYHC | Ab11083 | Abcam, Cambridge, UK |  | 1:200 |
| PAX7 | AF7584 | Affinity, Sterling, VA, USA | 1:1000 | 1:200 |
| MYOD | 18943-1-AP | Proteintech, Wuhan, China | 1:2000 | 1:200 |
| MYF5 | DF3089 | Affinity, Sterling, VA, USA | 1:1000 | 1:200 |
| PCNA | ab18197 | Abcam, Cambridge, UK | 1:1000 |  |
| Goat anti-mouse IgG | bs-0296G-HRP | Bioss, Beijing China | 1:10000 |  |
| Goat anti-Rabbit IgG | 31460 | Invitrogen, Waltham, CA, USA | 1:10000 |  |
| Goat anti-mouse IgG | ab96874 | Abcam, Cambridge, UK |  | 1:200 |
| Goat anti-Rabbit IgG | ab96883 | Abcam, Cambridge, UK |  | 1:200 |
